# Ascending aortic wall degeneration in patients with bicuspid versus tricuspid aortic valve

**DOI:** 10.1186/s13019-022-01864-0

**Published:** 2022-05-07

**Authors:** Ari Mennander, Ivana Kholova, Saku Pelttari, Timo Paavonen

**Affiliations:** 1 Tampere University Heart Hospital and Tampere University Medical School, SDSKIR, Elämänaukio 1, P.O. Box 2000, 33521 Tampere, Finland; 2 Department of Pathology, Fimlab Laboratories, Tampere University Hospital and Tampere University Medical School, Tampere, Finland

**Keywords:** Aortic wall degeneration, Ascending aorta, Bicuspid aortic valve

## Abstract

**Background:**

The magnitude of ascending aortic degeneration in patients with bicuspid aortic valves (BAV) is controversial.

**Methods:**

The aim of this study was to investigate ascending aortic wall degeneration in patients with BAV as compared with tricuspid aortic valves (TAV). The ascending aortic wall of 67 consecutive patients was processed for histology and immunohistochemistry. The extent of surgery and wall degeneration were investigated. Unadjusted survival was evaluated by Kaplan–Meier analysis. Median follow-up for patients with BAV and TAV was 3.8 years (interquartile range [IQR] 3.5–4.1) and 3.7 years (IQR 3.4–3.9), respectively.

**Results:**

There were 33 patients with BAV and 34 with TAV. Mid-ascending aorta diameter was 54 mm (IQR 50–60). Replacement of the aortic valve, together with an ascending aortic prosthesis, was more frequent in BAV vs TAV patients (24% vs. 3%, *P* = 0.013). However, medial fibrosis, elastic fiber thinning, incremental medial degeneration and smooth muscle cell nuclei loss were less prominent in BAV vs TAV patients (0.1 ± 0.4 vs. 0.8 ± 1.4, *P* = 0.016; 0.6 ± 1.4 vs. 1.6 ± 2.0, *P* = 0.027; 1.7 ± 0.7 vs. 2.2 ± 0.8, *P* = 0.045 and 2.3 ± 1.5 vs. 3.2 ± 1.3, *P* = 0.026, respectively).

**Conclusions:**

Since degeneration of the ascending aortic wall was seldom prominent, histopathology alone may not support the need for surgery of the dilated ascending aorta in BAV patients as compared with TAV patients.

## Introduction

Bicuspid aortic valve (BAV) is present in 1–2% of the whole population [1–3]. It has been suggested that patients with BAV are genetically susceptible to early aortic events and poor outcome [2], a conception mainly deduced from circulatory blood flow pattern changes of the ascending aorta [3]. Current recommendations suggest surgery of the ascending aorta without elastopathy, when the aortic root or the ascending aortic diameter exceeds 55 mm [4]. In cases of BAV, surgery of the ascending aorta is also considered (a) if the aortic root or the ascending aortic diameter exceeds 50 mm in the presence of risk factors such as coarctation of the aorta, systemic hypertension, family history of dissection, or an increase in aortic diameter up to 0.3 mm/year, or (b) if the aortic root or the ascending aortic diameter exceeds 45 mm and a surgical aortic valve replacement is scheduled [4]. However, the association of aortic wall degeneration with BAV and outcome after surgery remains controversial [5–8].

The Consensus statement on surgical pathology of the aorta from the Society for Cardiovascular Pathology and the Association for European Cardiovascular Pathology was recently launched to clarify the nomenclature and diagnostic criteria of degeneration [9]. The Consensus statement describes detailed means to investigate degenerative aortic wall changes pertinent to the development of an ongoing aortic disease. As the ultimate aim of surgery for the ascending aorta is to prevent aortic events, the aim of this study was to investigate the presence and significance of ascending aortic wall degeneration in BAV patients as compared with tricuspid aortic valve (TAV) patients undergoing surgery for the ascending aorta in a single-center patient cohort.

## Methods

### Study protocol and surgery

After institutional review board approval (Ethical Committee of the Tampere University Hospital, Tampere, Finland, R15013), the need for informed consent was waived and the study conforms to the ethical guidelines of the Declaration of Helsinki. The ascending aortic wall resection of 67 consecutive patients undergoing surgery for dilatation of ascending aorta was obtained and processed for histology. Ascending aortic aneurysm was preoperatively confirmed and evaluated with computer tomography (CT). According to our Institutional policy, aortic aneurysm included an aortic diameter more than 5.0–5.5 cm wide or aortic growth more than 1 cm in a year. This definition was adjusted to the presence of Marfan syndrome, sex, patient size and symptoms according to The Yale Center criteria [10]. Surgery was performed between December 2006 and August 2012.

The decision on the extension of resection and surgical technique was at the discretion of the operating surgeon. When aortic aneurysm, including the sinotubular junction (STJ), was estimated as the reason for aortic regurgitation, STJ was tailored for a suitable graft in a supracoronary fashion. Whenever dilatation included the aorta root, a radical resection of the dilated ascending aorta, together with the root and the aortic valve, was performed. The graft size was estimated by the principal surgeon. Since the surgical procedure was performed upon surgical decision, the sample was procured from the middle of the resected area of the ascending aorta at the vicinity of STJ including the intact aortic wall.

### Histology and immunohistochemistry

**Fig. 1:**
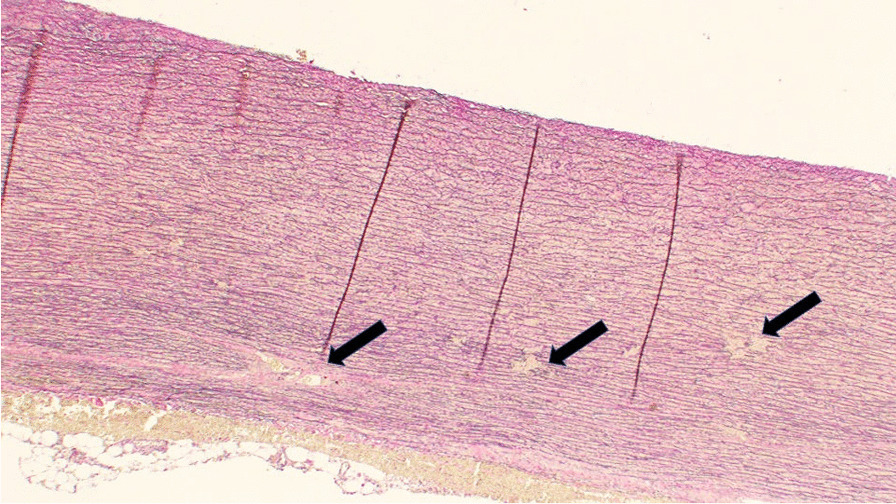
Representative histology of ascending aortic wall histology showing disruptions of elastic laminae indicative for degenerative medial layer (black arrows)

Two to five blocks of resected intact ascending aorta were embedded in paraffin, cut to 4 µm thick segments and stained with Hematoxylin and Eosin, Verhoeff-van Gieson, Elastase-van Gieson and Periodic Acid-Schiff. A representative, 1-cm long piece of ascending aortic wall corresponding to all different staining was evaluated systematically for all resected samples procured during surgery (Fig. 1).

Aortic wall histology and immunohistochemistry was performed using Ventana Lifesciences Benchmark XT© Staining module for leukocytes, T- and B-lymphocytes, plasma cells, macrophages, smooth muscle cells, cell proliferation, elastase and van Gieson staining. Ventana Lifesciences Antibody Dilution Buffer© was utilized for dilution media. The heights of different layers (adventitia, media and intima) were calculated for each sample [11].

### Quantification of medial degeneration

Medial degeneration of the ascending aorta was assessed by quantifying 11 different variables describing medial and adventitial damage [9, 12]. These included medial fibrosis, elastic fiber disorganization, elastic fiber loss/fragmentation, elastic fiber thinning, laminar medial collapse, classification of medial degeneration, mucoid extracellular matrix accumulation, smooth muscle cell disorganization, smooth muscle cell nuclei loss and medial thickness of vasa vasorum, and adventitial fibrosis. According to the consensus, the variables describing medial degeneration were categorized as none, mild, moderate and severe on a scale of 0–3 [9].

### Follow-up protocol

Documentation of mortality and morbidity was available for all the patients. For the included study patients, follow-up consisted of physical examination and echocardiography at three months after surgery, and on-demand thereafter including computed tomography. Morbidity after surgery included documentation of concomitant coronary artery bypass grafting, pacemaker implantation, cerebral stroke, reoperation, and aortic event.

### Statistical analysis

Continuous variables were expressed as medians including interquartile (IQR), incremental variables of media layer degeneration as means with standard deviations, and were compared using the Mann–Whitney test. Categorical variables were presented as numbers and percentages, and were compared using χ^2^ or Fisher’s exact tests. In order to seek clinical relevance associated with immunohistochemistry, the patients were divided into two groups in accordance with the presence of either BAV or TAV. Unadjusted survival was evaluated by Kaplan–Meier analysis with log-rank tests. All analyses were conducted using the IBM SPSS Statistics version 26.0 (IBM Corporation, Armonk, NY, USA) and R 4.1.2 statistical software (R Foundation for Statistical Computing, Vienna, Austria) with *P* < 0.05 as the criterion for significance.

## Results

### Patient characteristics

Patient characteristics are shown on Table 1. There were 33 patients with BAV and 34 with TAV. The median age for the patients was 66 years (IQR 56–71). Hypertension was frequent in patients with TAV as compared with BAV patients (*P* = 0.001). The median aortic diameter was 53 mm (IQR 50–57) in BAV patients and 55 mm (IQR 52–60) in TAV patients (*P* = 0.042). Median follow-up for patients with BAV and TAV was 3.8 years (IQR 3.5–4.1) and 3.7 years (IQR 3.4–3.9), respectively.

**Table 1:** Patient characteristics

	All patients	BAV	TAV	*P* value
Number of patients	67	33	34	
Age (years, interquartile)	66 (56–71)	64 (55–72)	68 (61–71)	0.133
Male, n	50 (75%)	26	24	0.576
Hypertension, n	44 (68%)	15	29	0.001
Diabetes, n	9 (14%)	3	6	0.475
Hypercholesterolemia, n	17 (26%)	11	6	0.166
Vasculitis, n	6 (8%)	1	5	0.198
Arthritis	6 (9%)	2	4	0.673
Asthma, n	4 (6%)	0	4	0.114
Myocardial coronary artery disease, n	14 (21%)	7	7	1
History of stroke	4 (6%)	2	2	1
Earlier abdominal aorta aneurysm surgery	2 (3%)	0	2	0.492
Mid-ascending aorta diameter (mm, interquartile)	54 (50–60)	53 (50–57)	55 (52–60)	0.042
Moderate to severe aortic valve regurgitation, n	36 (56%)	14	22	0.139

BAV = bicuspid aortic valve; TAV = tricuspid aortic valve

### Operative technique

The operative technique is shown on Table 2. A mechanical valve was implanted with or without a conduit prosthesis in 11 out of 33 (33%) BAV patients as opposed to only 4 out of 34 (12%) TAV patients owing to patient age. Altogether, replacement of the aortic valve, together with an ascending aortic prosthesis, but without replacement of the aortic root, was more frequent in BAV vs TAV patients (24% vs 3%, *P* = 0.013). The aortic valve, the root and the ascending aorta were replaced using a conduit prosthesis in 55% BAV versus 71% TAV patients (*P* = 0.212). The ascending aorta only was replaced in 16 patients. Concomitant coronary artery bypass grafting was performed in four BAV and three TAV patients.

**Table 2:** Operative details according to surgical evaluation of extension of diseased aorta

	All patients	BAV	TAV	*P* value
	67	33	34	
Graft replacement of root and ascending aorta				
Mechanical conduit	10 (15%)	6 (9%)	4 (6%)	0.512
Biological conduit	32 (48%)	12 (18%)	20 (30%)	0.088
Graft replacement of ascending aorta				
Mechanical valve + prosthesis	**5 (8%)**	**5 (8%)**	**0**	**0.025**
Biological valve + prosthesis	4 (6%)	3 (5%)	1 (1%)	0.356
Prosthesis	16 (24%)	7 (11%)	9 (13%)	0.776

BAV = bicuspid aortic valve; TAV = tricuspid aortic valve

Significant differences between groups are in bold

### Perioperative findings, histology and immunohistochemistry

As shown on Table 3, medial fibrosis remained less significant in BAV as compared with TAV patients (0.1 ± 0.4 vs. 0.8 ± 1.4, *P* = 0.016, respectively). Elastic fiber thinning was less present in BAV versus TAV patients (0.6 ± 1.4 vs. 1.6 ± 2.0, *P* = 0.027). Incremental medial degeneration was 1.7 ± 0.7 in BAV as compared with 2.2 ± 0.8 in TAV patients (*P* = 0.045). There was a tendency for decreased mucoid extracellular matrix accumulation in BAV as compared with TAV patients (4.3 ± 0.9 vs. 4.9 ± 1.2, *P* = 0.051). Smooth muscle cell nuclei loss was less prominent in BAV as compared with TAV patients (2.3 ± 1.5 vs. 3.2 ± 1.3, *P* = 0.026).

**Table 3:** Histology and quantitative immunohistochemistry

	All patients	BAV	TAV	*P* value
Adventitial fibrosis	0.2 ± 0.4	0.1 ± 0.3	0.2 ± 0.4	0.221
Medial fibrosis	**0.5 ± 1.1**	**0.1 ± 0.4**	**0.8 ± 1.4**	**0.016**
Elastic fiber disorganization	1.0 ± 1.0	0.8 ± 0.9	1.1 ± 1.1	0.191
Elastic fiber loss/fragmentation	3.6 ± 1.4	3.3 ± 1.2	3.8 ± 1.6	0.206
Elastic fiber thinning	**1.1 ± 1.8**	**0.6 ± 1.4**	**1.6 ± 2.0**	**0.027**
Laminar medial collapse	0.6 ± 1.3	0.3 ± 0.9	0.8 ± 1.5	0.184
Classification of medial degeneration	**1.9 ± 0.8**	**1.7 ± 0.7**	**2.2 ± 0.8**	**0.045**
Mucoid extracellular matrix accumulation	4.6 ± 1.2	4.3 ± 0.9	4.9 ± 1.2	0.051
Smooth muscle cell disorganization	0.6 ± 0.8	0.4 ± 0.7	0.8 ± 0.9	0.121
Smooth muscle cell nuclei loss	**2.8 ± 1.4**	**2.3 ± 1.5**	**3.2 ± 1.3**	**0.026**
Medial thickness of vasa vasorum	0.2 ± 0.4	0.3 ± 0.5	0.2 ± 0.4	0.407

Mean ± standard deviation; BAV = bicuspid aortic valve; TAV = tricuspid aortic valve

Significant differences between groups are in bold

### Morbidity

Two BAV patients received a pacemaker. During follow-up, one TAV patient had a cerebral stroke, and another TAV patient experienced a B-type aortic dissection. There were no reoperations during follow-up.

### Survival

**Fig. 2:**
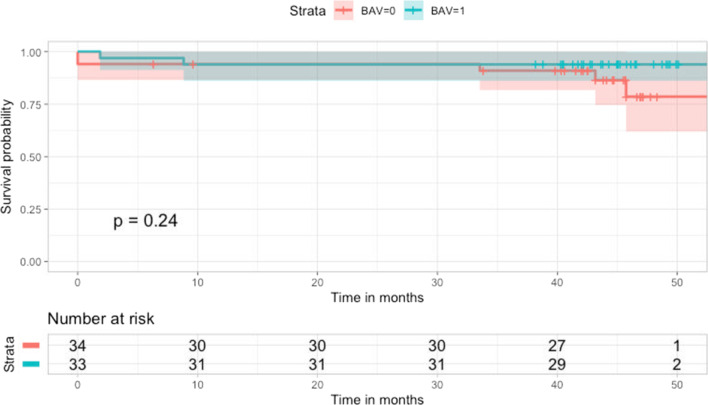
Survival probability (%) of patients after surgery for ascending aorta with bicuspid (*blue line*) and tricuspid aortic valve (*red line*). Time-varying outcome according to Kaplan–Meier estimation. *BAV* = *1*, bicuspid aortic valve; *BAV* = *0*, tricuspid aortic valve. Log rank *P* = 0.240

According to Kaplan–Meier analysis (Fig. 2), survival did not significantly differ between patients with BAV and TAV (log rank *P* = 0.240). Two BAV and three TAV patients died during follow-up. Of these, two patients with TAV died during hospitalization.

## Discussion

This study shows that less prominent ascending aortic wall degeneration with increased mid-ascending aorta diameter at a relatively younger age characterized patients with BAV as compared with TAV. The Consensus statement on surgical pathology of the aorta from the Society for Cardiovascular Pathology and the Association for European Cardiovascular Pathology provided a valuable diagnostic platform to evaluate the degree of aortic wall degeneration.

Patients undergoing surgery for the ascending aorta have a multifactorial presentation of clinical symptoms [7, 13]. Despite different patient characteristics, current treatment includes surgical resection of the dilated aortic portion together with surgery for the aortic valve whenever needed. The presence of degenerative histological features would further justify resection of the frail aorta. The detailed evaluation of degenerative aortic wall variables aids objective comparison of different aortic diseases associated with aortic valve morphology and may prevent unnecessary resection in those patients without frail aortic wall.

The almost 50% incidence of BAV in patients undergoing surgery for the ascending aorta seems high in this real-life cohort. However, relatively similar or even higher numbers of aortic surgeries have been reported earlier as many BAV patients undergoing aortic valve surgery have an at least 45 mm ascending aortic diameter [14, 15], a surgical strategy that complies with current recommendations [4]. Many BAV patients received a mechanical aortic valve prosthesis since BAV patients underwent aortic surgery at a relatively early age. The asymmetry of the BAV cusps is often reflected on the configuration of the ascending aorta; the aortic diameter may considerably differ depending on different projections during CT imaging [3]. A slightly increased asymmetric aortic diameter in BAV patients may not indicate prominent and consistent aortic wall degeneration.

Risk factors for aortic dilatation in general may include hypertension, male sex, family history of aortic aneurysm, and atrial fibrillation; in contrast, diabetes, smoking and coronary artery disease are more controversial risk factors for dilatation of the ascending aorta per se [16]. In our study, hypertension and aortic wall degeneration, were statistically more frequent in TAV patients than in those with BAV. The lack of significant difference in age between the patient groups prevents from further speculation whether absolute age correlates with medial degeneration in TAV patients, as previously suggested [15].

Indeed, aortic wall degeneration- as observed by medial fibrosis, elastic fiber thinning, incremental medial degeneration and smooth muscle cell loss of the tissue- was significantly less prominent in BAV as compared with TAV patients. This importantly confirms previous studies suggesting for the degenerative features of the ascending aorta found predominantly in TAV patients [6, 8, 15]. Dilatation of the mid-ascending aorta is not solely associated with the presence of bicuspid aortic valve or increased aortic wall degeneration.

## Conclusions

Ascending aortic dilatation is not associated with increased aortic wall degeneration in BAV patients as compared with TAV patients. The Consensus statement on surgical pathology of the aorta from the Society for Cardiovascular Pathology and the Association for European Cardiovascular Pathology provides an important diagnostic methodology to evaluate the degree of degeneration of the ascending aorta and may correct misconceptions related with aortic valve configuration. This study suggests that the same surgical strategy of resection of the dilated ascending aorta may apply to both patients with either BAV or TAV.

### Limitations

This study represents a real-life single-center contemporary cohort. The limitations of this study include the small number of patients with a relatively short follow-up, and aortic wall histology is obviously only available in patients that underwent surgery.

## Data Availability

The datasets used and analyzed during the current study are available from the corresponding author on reasonable request.

## Abbreviations

BAV: Bicuspid aortic valve
IQR: Interquartile range
TAV: Tricuspid aortic valve

## References

[1] Sillesen A-S, Vøgg O, Pihl C, Raja AA, Sunberg K, Vedel C, Zingenberg H, Jørgensen FS, Vejlstrup N, Iversen K, Bundgaard H (2021). Prevalence of bicuspid aortic valve and associated aortopathy in newborns in Copenhagen, Denmark. JAMA.

[2] Michelena HI, Khanna AD, Mahoney D, Margaryan E, Topilsky Y, Suri RM, Eidem E, Edwards WD, Sundt TM, Enriquez-Sarano M (2011). Incidence of aortic complications in patients with bicuspid aortic valves. JAMA.

[3] Youssefi P, Gomez A, He T, Anderson L, Bunce N, Sharma R, Figueroa CA, Jahangiri M (2017). Patient-specific computational fluid dynamics—Assessment of aortic hemodynamics in a spectrum of aortic valve pathologies. J Thorac Cardiovasc Surg.

[4] The Task Force for the Diagnosis and Treatment of Aortic Diseases of the European Society of Cardiology (ESC). 2014 ESC Guidelines on the Diagnosis and Treatment of Aortic Diseases: Document Covering Acute and Chronic Aortic Diseases of the Thoracic and Abdominal Aorta of the Adult. The Task Force for the Diagnosis and Treatment of Aortic Diseases of the European Society of Cardiology (ESC). Eur Heart J. 2014;1:2873–2926. 10.1093/eurheartj/ehu281 25173340

[5] Milewski RK, Habertheuer A, Bavaria JE, Siki M, Szeto WY, Krause E, Korutla V, Desai ND, Vallabhajosyula P (2017). Fate of remnant sinuses of Valsalva in patients with bicuspid and trileaflet valves undergoing aortic valve, ascending aorta, and aortic arch replacement. J Thorac Cardiovasc Surg.

[6] Leone O, Corsini A, Pacini D, Corti B, Lorenzini M, Laus V, Foa A, Reggiani MLB, Di Marco L, Rapezzi C (2019). The complex interplay among atherosclerosis, inflammation, and degeneration in ascending thoracic aortic aneurysms. J Thorac Cardiovasc Surg.

[7] Kanekoa T, Shekara P, Ivkovica V, Longfordb NT, Huangc C-C, Sigurdsson MI, Neely RC, Yammine M, Ejiofor JI, Vieira VM, Shahram JT, Habchi KM, Malzberg GW, Martin PS, Bloom J, Isselbacher EM, Muehlschlegel JD, Bicuspid Aortic Valve Consortium (BAVCon), Sundt 3^rd^ TM. Should the dilated ascending aorta be repaired at the time of bicuspid aortic valve replacement? Eur J Cardio-Thorac Surg. 2018;53:560–568. 10.1093/ejcts/ezx387 PMC6018902 29149323

[8] Heng E, Stone JR, Kim JB, Lee H, MacGillivray TE, Sundt TM (2015). Comparative histology of aortic dilatation associated with bileaflet versus trileaflet aortic valves. Ann Thorac Surg.

[9] Halushka MK, Angelini A, Bartoloni G, Basso C, Batoroeva L, Bruneval P, Buja LM, Butany J, d’Amati G, Fallon JT, Gallagher PJ, Gittenberger-de Groot AC, Gouveia RH, Kholova I, Kelly KL, Leone O, Litovsky SH, Maleszewski JJ, Miller DV, Mitchell RN, Preston SD, Pucci A, Radio SJ, Rodriguez ER, Sheppard MN, Stone JR, Suvarna SK, Tan CD, Thiene G, Veinot JP, van der Wal AC. Consensus statement on surgical pathology of the aorta from the Society for Cardiovascular Pathology and the Association for European Cardiovascular Pathology: II. Noninflammatory degenerative diseases - nomenclature and diagnostic criteria. Cardiovasc Pathol. 2016;25:247–257. 10.1016/j.carpath.2016.03.002 27031798

[10] Elefteriades JA (2008). Thoracic aortic aneurysm: reading the enemy’s playbook. World J Surg.

[11] Levula M, Paavonen T, Valo T, Pelto-Huikko M, Laaksonen R, Kahonen M, Huovila A, Lehtimaki T, Tarkka M, Mennander AA (2011). A disintegrin and metalloprotease -8 and -15 and susceptibility for ascending aortic dissection. Scand J Clin Lab Invest.

[12] Stone JR, Bruneval P, Angelini A, Bartoloni G, Basso C, Batoroeva L, Buja LM, Butany J, d’Amati G, Fallon JT, Gittenberger-de Groot AC, Gouveia RH, Halushka MK, Kelly KL, Kholova I, Leone O, Litovsky SH, Maleszewski JJ, Miller DV, Mitchell RN, Preston SD, Pucci A, Radio SJ, Rodriguez ER, Sheppard MN, Suvarna SK, Tan CD, Thiene G, van der Wal AC, Veinot JP (2015). Consensus statement on surgical pathology of the aorta from the Society for Cardiovascular Pathology and the Association for European Cardiovascular Pathology: I. Inflammatory diseases. Cardiovasc Pathol.

[13] Kirsch EW, Radu NC, Gervais M, Allaire E, Loisance DY (2006). Heterogeneity in the remodeling of aneurysms of the ascending aorta with tricuspid aortic valves. J Thorac Cardiovasc Surg.

[14] Hui SK, Fan C-PS, Christie S, Feindel CM, David TE, Ouzounian M (2018). The aortic root does not dilate over time after replacement of the aortic valve and ascending aorta in patients with bicuspid or tricuspid aortic valves. J Thorac Cardiovasc Surg.

[15] Karalko M, Stejskal V, Dergel M, Gofus J, Timbilla S, Zaloudkova L, Zacek P, Pojar M, Vojacek J (2021). Histopathological changes in dilated ascending aorta associated with aortic valve cuspidity. Eur J Cardio-Thorac Surg.

[16] Obel LM, Diederichsen AC, Steffensen FH, Frost L, Lambrechtsen J, Busk M, Urbonaviciene G, Egstrup K, Karon M, Rasmussen LM, Gerke O, Bovling AS, Lindholt JS (2021). Population-based risk factors for ascending, arch, descending, and abdominal aortic dilatations for 60–74-year-old individuals. JACC.

